# Gray Matter Alterations in Post-Traumatic Stress Disorder, Obsessive–Compulsive Disorder, and Social Anxiety Disorder

**DOI:** 10.3389/fnbeh.2015.00219

**Published:** 2015-08-20

**Authors:** Bochao Cheng, Xiaoqi Huang, Shiguang Li, Xinyu Hu, Ya Luo, Xiuli Wang, Xun Yang, Changjian Qiu, Yanchun Yang, Wei Zhang, Feng Bi, Neil Roberts, Qiyong Gong

**Affiliations:** ^1^Department of Radiology, Huaxi MR Research Center, West China Hospital of Sichuan University, Chengdu, China; ^2^Department of Psychiatry, West China Hospital of Sichuan University, Chengdu, China; ^3^Department of Oncology, West China Hospital of Sichuan University, Chengdu, China; ^4^Clinical Research Imaging Centre, School of Clinical Sciences, University of Edinburgh, Edinburgh, UK

**Keywords:** gray matter volume, post-traumatic stress disorders, obsessive–compulsive disorder, social anxiety disorder

## Abstract

Post-traumatic stress disorder (PTSD), obsessive–compulsive disorder (OCD), and social anxiety disorder (SAD) all bear the core symptom of anxiety and are separately classified in the new DSM-5 system. The aim of the present study is to obtain evidence for neuroanatomical difference for these disorders. We applied voxel-based morphometry (VBM) with Diffeomorphic Anatomical Registration Through Exponentiated Lie to compare gray matter volume (GMV) in magnetic resonance images obtained for 30 patients with PTSD, 29 patients with OCD, 20 patients with SAD, and 30 healthy controls. GMV across all four groups differed in left hypothalamus and left inferior parietal lobule and *post hoc* analyses revealed that this difference is primarily due to reduced GMV in the PTSD group relative to the other groups. Further analysis revealed that the PTSD group also showed reduced GMV in frontal lobe, temporal lobe, and cerebellum compared to the OCD group, and reduced GMV in frontal lobes bilaterally compared to SAD group. A significant negative correlation with anxiety symptoms is observed for GMV in left hypothalamus in three disorder groups. We have thus found evidence for brain structure differences that in future could provide biomarkers to potentially support classification of these disorders using MRI.

## Introduction

Anxiety disorders are one of the most prevalent categories of psychopathology (Kessler et al., 2005). They have long been documented to possess comorbidities (Andrews et al., 2008; Watson et al., 2008; Al-Asadi et al., 2014) and share features of excessive, irrational fear and anxiety, related behavior abnormality, and avoidance of anxiety triggers (Storch et al., 2008). The comorbidity not only hampers treatment (Gershuny et al., 2002) but also increases the risk of suicide in patients with these conditions on account of significant differences being appreciated diagnosed (Albert et al., 2008). However, until recently very few studies have attempted to determine the differences between these anxiety disorders from a neuroimaging perspective.

The previous Diagnostic and Statistical Manual of Mental Disorders IV (DSM-IV) system classified all the following as anxiety disorders, namely panic disorder, post-traumatic stress disorder (PTSD), phobic disorders (i.e., social anxiety disorder (SAD), specific phobias, and agoraphobia), generalized anxiety disorder (GAD), and obsessive–compulsive disorder (OCD). However, although they share many features with other anxiety disorders, such as excessive fear, avoidance, and hyperarousal (Storch et al., 2008; Friedman et al., 2011) according to the new DSM-5 system, PTSD and OCD are no longer included in the anxiety disorder category.

Nevertheless, the first-line of medication that is typically recommended for PTSD, OCD, and the majority of other anxiety disorders, such as SAD and GAD, are still selective serotonin reuptake inhibitors (SSRIs) (Bandelow et al., 2008). Huppert et al. (2005) found evidence for an overlap of symptoms that contribute to the diagnostic overlap of OCD and PTSD and Gren-Landell et al. (2013) also found similarities between SAD and PTSD in the form of recurrent memories and intrusive and distressing images of earlier aversive events. Furthermore, a combined study of PTSD, OCD, and SAD revealed that treatment with SSRIs produced effects in a common neurocircuitry between all the patients groups (Carey et al., 2004). Thus, despite these clinical similarities the potential neural mechanisms underlying these disorders might be different. Neuroimaging has the potential to identify this neural substrate, elucidate potential significant differences, and indeed to determine the neurobiological bases of psychiatric disorders in general (Paulus, 2008). Consequently, many neuroimaging studies have been performed to measure brain structure and function in patients with various anxiety-related disorders. Specifically, gray matter volume (GMV) has been measured on a voxel by voxel basis using a technique known as voxel-based morphometry (VBM) study because it represents an important substrate of neural functioning and its reduction is a sensitive indicator of brain abnormality (Ashburner and Friston, 2001).

Although many structural magnetic resonance (MR) imaging studies have been performed of PTSD and OCD, there has been few of SAD. In PTSD, widespread gray matter abnormalities have been revealed, including anterior cingulate cortex (ACC), hippocampal structures, insula, and prefrontal cortex (Hull, 2002; Abe et al., 2006). For example, using VBM Herringa et al. (2012) reported a reduction of GMV in unmedicated combat veterans PTSD in subgenual ACC, caudate, hypothalamus, insula, and left temporal cortex, and Kasai et al. (2008) reported GMV loss only in patients with combat-related PTSD in pregenual AC. Reduced bilateral hippocampal volume was found in adults with childhood maltreatment-related PTSD compared to healthy controls (HCs) (Woon and Hedges, 2008), and a recent meta-analysis of VBM studies reported GMV reduction to be most robust in medial prefrontal cortex in PTSD compared to that of both trauma-exposed controls without PTSD or non-traumatized HCs (Li et al., 2014). With regard to OCD, anomalies in a prefrontal-basal ganglia network used to be considered the main pathophysiology of this disorder and this was supported by reports of reduced GMV in frontal cortex and increased GMV in striatum regions (Pujol et al., 2004; Rotge et al., 2010). However, a recent meta-analysis showed gray matter differences extending beyond frontal and striatal regions to include thalamus, parietal, and cerebellar regions (Eng et al., 2015). Only one VBM study has been performed in patients with SAD and the reported findings are quite different to the results of all the above-mentioned studies of PTSD and OCD. In particular, a decrease in GMV is reported in left lateral orbitofrontal cortex (OFC) and bilateral temporal lobes and an increase in left parahippocampal gyrus and middle occipital cortex, bilateral supramarginal gyrus and angular cortices, and left cerebellum (Talati et al., 2013).

Understanding of the functional neuroanatomy of anxiety disorders has benefited greatly from the development of functional neuroimaging techniques. One of the earliest studies used positron emission tomography (PET) to measure regional cerebral blood flow (rCBF) and led to the suggestion that parts of the paralimbic belt together with right inferior frontal cortex and subcortical nuclei mediate symptoms across the three anxiety disorders of PTSD, OCD, and simple phobia (Rauch et al., 1997). More recently, a meta-analysis compared the findings from fMRI and PET studies of PTSD, SAD, and specific phobia, and reported a unique effect in patients with PTSD who showed consistently greater activity than matched comparison subjects in the amygdala and insula, structures linked to negative emotional responses (Etkin and Wager, 2007).

Only a few studies have specifically sought to investigate whether there are potentially significant differences in neuroanatomical structure between patients with different anxiety disorders. For example, Radua (2010) performed a meta-analysis of VBM studies so as to compare findings in OCD relative to other anxiety disorders and reported increased GMV in bilateral lenticular and caudate nuclei of OCD patients, and reduced GMV in left lenticular nuclei in patients with other anxiety disorders (mainly panic and PTSDs). However, as far as we are aware, no direct comparison of potential differences in brain structure in patients with PTSD, OCD, and SAD has yet been performed. Given the limitations of meta-analyses, including difficulty in obtaining raw data (Salimi-Khorshidi et al., 2009), a direct comparison is necessary to clarify the neural differences between these disorders.

In the present study, we recruited 30 patients with PTSD, 29 patients with OCD, and 20 patients with SAD as well as 30 HCs from the same geographic location and used an identical MRI system and MR imaging protocol to obtain high-resolution 3D images of the brain. Our aim was to apply a state of art whole brain VBM analysis incorporating the Diffeomorphic Anatomical Registration Through Exponentiated Lie (DARTEL) algorithm (Ashburner and Friston, 2001) to determine if the three patient groups showed common neuroanatomical differences in GMV compared to matched controls, as well as to identify any disorder-specific effects. As discussed, VBM overcomes the limitations of ROI-based analyses and DARTEL optimizes the sensitivity of the analysis compared to standard VBM (Klein et al., 2009). With regard to disorder-specific effects, given that PTSD is known to be related to stress, which itself can also cause functional (Lui et al., 2009) and structural (Chen et al., 2013) neuroanatomical changes, we predicted that PTSD would show greater and more extensive GM alterations compared with the other two disorders.

Classification of patients was based on the DSM-IV in which PTSD, OCD, and SAD were all classified as anxiety disorders. DSM-IV has, however, subsequently been revised to DSM-5 in which PTSD and OCD are separated from the group of anxiety disorders which still includes SAD. The present study thus fortuitously provided an opportunity to investigate whether there are neuroanatomical findings that support the revision in the classification of anxiety disorders between DSM-IV and DSM-5.

## Materials and Methods

### Study design and participants

This study was approved by the local Research Ethics Committee (REC) and was conducted in accordance with the Declaration of Helsinki, consistent good clinical practice (GCP), and applicable regulatory requirements. All participants with PTSD, OCD, and SAD as well as HCs provided fully informed written consent.

All participants were right-handed and of Han Chinese ethnicity. Patients were recruited from the Mental Health Center of West China Hospital and diagnosed by two attending psychiatrists using the Structured Clinical Interview for the DSM-IV (SCID) criteria. Exclusion criteria included the presence of neurologic diseases, a history of head injury, alcohol or drug abuse, mental retardation, and all other DSM-IV Axis I disorders. In addition, the 17-item Hamilton Rating Scale for Depression (HAM-D) (Hamilton, 1967) and the Hamilton Rating Scale for Anxiety (HAM-A) (Hamilton, 1959) were used to rate the severity of participants’ depressive and anxiety symptoms prior to their MRI scans.

The PTSD group consisted of 30 survivors (Table 1) of the Wenchuan Earthquake who had not received treatment (Table 1). These patients were interviewed and screened using the PTSD checklist (PCL) (Ruggiero et al., 2003) and the Clinician-Administered PTSD Scale (CAPS) (Blake et al., 1995). We also recruited 29 patients with OCD (Table 1) in whom the Yale–Brown Obsessive–Compulsive Scale (Y–BOCS) and the clinician-rated Yale–Brown Obsessive–Compulsive Scale symptom checklist were used to assess symptoms (Goodman et al., 1989). Of the 29 patients with OCD, 15 were drug-naive, whereas 14 had received typical medications for OCD symptoms with different illness durations. However, all patients had experienced at least 2-week medication cessation period before MR scanning. Twenty patients with SAD who had never received any treatment (Table 1) were evaluated with the Liebowitz Social Anxiety Scale (LSAS). Patients who presented with other psychiatric disorders according to the SCID were excluded from the sample.

**Table 1:** **Demographic data and clinical characteristics**.

Characteristics	Patient groups						HCs (M/F: 21/9)	
	OCD (M/F: 20/9)		PTSD (M/F: 21/9)		SAD (M/F: 13/7)			
	**Mean**	**SD**	**Mean**	**SD**	**Mean**	**SD**	**Mean**	**SD**
Age (years)	24.2	7.7	26.3	8.1	23.3	3.7	26.2	6.6
Disease duration (months)*	66.2	65.0	12.5	2.7	47.9	44.1	–	–
HAM-A*	8.1	2.3	10.9	2.3	6.2	4.8	–	–
HAM-D*	9.6	2.6	14.2	5.3	8.8	8.5	–	–
Y-BOCS	23.4	5.2	–	–	–	–	–	–
PCL	–	–	46.9	12.5	–	–	–	–
CAPS	–	–	56.4	13.6	–	–	–	–
LSAS (fear factor)	–	–	–	–	27.8	6.9	–	–
LSAS (avoidance factor)	–	–	–	–	24.9	8.4	–	–
LSAS (total score)	–	–	–	–	52.8	13.7	–	–

*Significant differences in disease duration, HAM-A, and HAM-D scores among the three anxiety sub-groups (p < 0.05).

*CAPS, clinician-administered PTSD scale; HAM-A, Hamilton Rating Scale for anxiety; HAM-D, Hamilton Rating Scale for Depression; HC, healthy control; LSAS, Liebowitz Social Anxiety Scale; M/F, male/female; OCD, obsessive–compulsive disorder; PCL, PTSD checklist; PTSD, post-traumatic stress disorder; SAD, social anxiety disorder; SD, standard deviation; Y-BOCS, Yale–Brown Obsessive–Compulsive Scale*.

Thirty HCs, who were matched for age, gender, and education level with the patients, were recruited from the local area via poster advertisements. HCs were screened using the SCID non-patient version (SCID-NP) to exclude those with a history of psychiatric illness.

### Image acquisition

All participants were scanned at the Huaxi MR Research Center (HMRRC) using a dedicated 3-T MRI system (EXCITE; General Electric, US; 8-channel head coil). High-resolution T1-weighted images were acquired using a volumetric 3D spoiled gradient recall (SPGR) sequence (repetition time = 8.5 ms, echo time = 3.4 ms, flip angle = 12°, slice thickness = 1.0 mm, field of view = 240 mm × 240 mm, voxel size = 0.47 mm × 0.47 mm × 1 mm) with an eight-channel phase array head coil that produces 156 contiguous axial slices to scan the entire brain. Foam padding and earplugs were used to reduce head motion and scanner noise.

### VBM-DARTEL preprocessing

The 3D T1-weighted MR image data were analyzed using Statistical Parametric Mapping 8 software (SPM8; Welcome Department of Imaging Neuroscience, London, England)^1^ running in MATLAB 7.11 (Mathworks, Natick, MA, USA). During the VBM preprocessing step, DARTEL was used to improve inter-subject co-registration of structural MR images. The images for each participant were inspected for artifacts, and subsequently transformed to Montreal Neurological Institute (MNI) space. Next, the images were segmented into gray matter, white matter, and cerebrospinal fluid. The segmented images of gray matter, white matter, and cerebrospinal fluid were roughly aligned for all 109 participants. Subsequently, a DARTEL template was constructed with each voxel resampled to 1.5 mm × 1.5 mm × 1.5 mm. The warped data were smoothed with an 8-mm full width at half maximum (FWHM).

### Statistical analysis

Comparison of the demographic and clinical variables between groups was performed with ANOVA using statistical software SPSS (version 17.0). VBM was performed using the DARTEL algorithm in SPM8 to quantify GMV volume. Group differences in GMV were assessed using an analysis of covariance (ANCOVA) with whole-brain volume, age, and gender used as covariates and subsequent *post hoc* analysis used a two-sample *t*-test with whole-brain volume, age, gender, disease duration, and symptom severity as covariates. First, a whole-brain analysis of group differences in GMV was performed using the general linear model with a single-factor ANCOVA design that included four independent groups. This approach resulted in a map of the brain areas that demonstrated significant differences in GMV across the anxiety disorder and HC groups. Second, GMV was compared across groups. Significance was set at a value of *p* < 0.05, following a family-wise error (FWE) correction for multiple comparisons with a minimum cluster size of 70 voxels. Statistical maps were overlaid on the DARTEL templates using MRIcro software^2^. The results are presented based on the voxel of peak significance.

Finally, an ROI analysis was performed. In particular, ROIs consisting of those voxels in the regions showing the greatest significant differences (P_FWE-corrected_ < 0.05) among the four groups as well as between the patients with PTSD and HCs were defined. The ROIs were selected using the xjView tool in SPM8. GMV was extracted for the ROI’s using the MarsBar toolbox^3^. The results were analyzed using SPSS. First, a *post hoc* analysis of the homogeneous subset was used to determine significant differences between groups; *p* < 0.05 after correction for multiple comparisons (FWE-corrected) was considered to be significant. Second, a correlation analysis was performed between GMV in the aforementioned ROIs and clinical variables, including HAM-A, HAM-D, PCL, CAPS score, and disease duration.

## Results

### Demographic data and clinical characteristics

A total of 109 participants were studied. Table 1 shows the demographic data and clinical characters of these 79 patients and 30 HCs. No significant differences were evident among the four groups with regard to age (*p* = 0.07) and gender (*p* = 0.99). Among the anxiety disorder sub-groups, significant differences were evident with regard to disease duration as well as HAM-A and HAM-D scores (Table 1).

### VBM-DARTEL analysis

**Figure 1:**
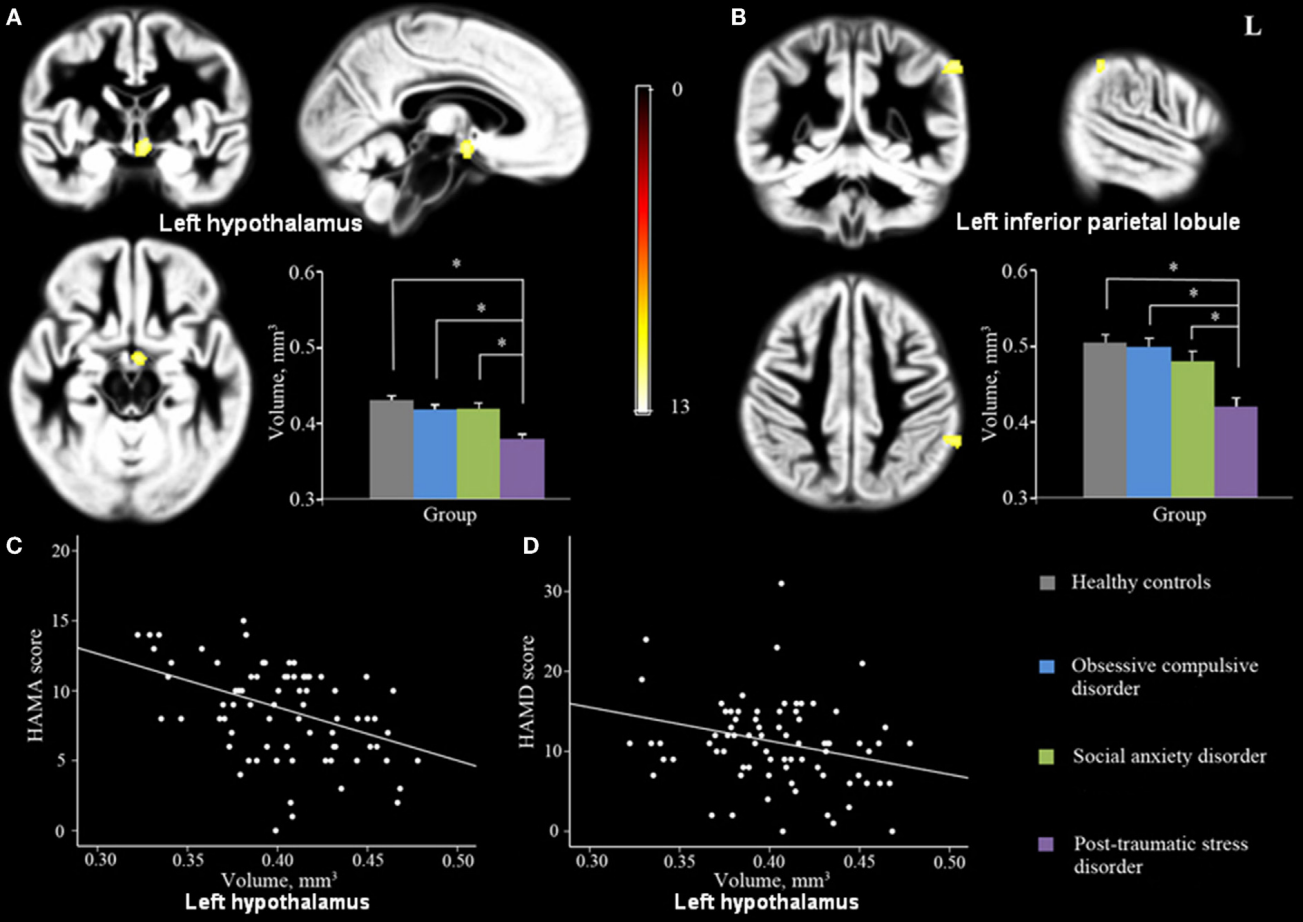
**Significant GMV differences were observed among PTSD, OCD, SAD and controls in left hypothalamus (A) and left IPL (B) shown projected onto the gray matter template built using DARTEL**. Significant correlations were also observed between GMV in left hypothalamus and HAM-A **(C)** and HAM-D **(D)** scores. (*P*_cluster-level_ < 0.05 with a minimum cluster size of 70 voxels after FWE correction for whole-brain volume, age, and gender). Abbreviations: GMV, gray matter volume; DARTEL, Diffeomorphic Anatomical Registration Through Exponentiated Lie; HAM-A, Hamilton Rating Scale for Anxiety; HAM-D, Hamilton Rating Scale for Depression; IPL, inferior parietal lobule.

GMV across the four groups differed significantly in left hypothalamus and left inferior parietal lobule (IPL; Figures 1A,B; Table 2). In addition, a *post hoc* analysis revealed that this difference was primarily due to differences between the PTSD group and the other groups (Figure 1). As shown in Figure 1, PTSD showed lower GMV in above-mentioned regions than all other three groups (*p* < 0.05).

**Table 2:** **Significant differences in GMV observed between the four groups**.

Anatomical region	Location	Cluster size	Test value		MNI coordinate		
			*p*_(cluster-level)_	F			
Hypothalamus	Left	78	0.012	11.91	−5	−4	−9
Inferior parietal lobule	Left	71	0.015	11.66	−57	−45	42

*p_(FWE-corrected)_ < 0.05*.

*ANCOVA, analysis of covariance; FWE, family-wise error; MNI, Montreal Neurological Institute*.

### HC versus PTSD

**Figure 2:**
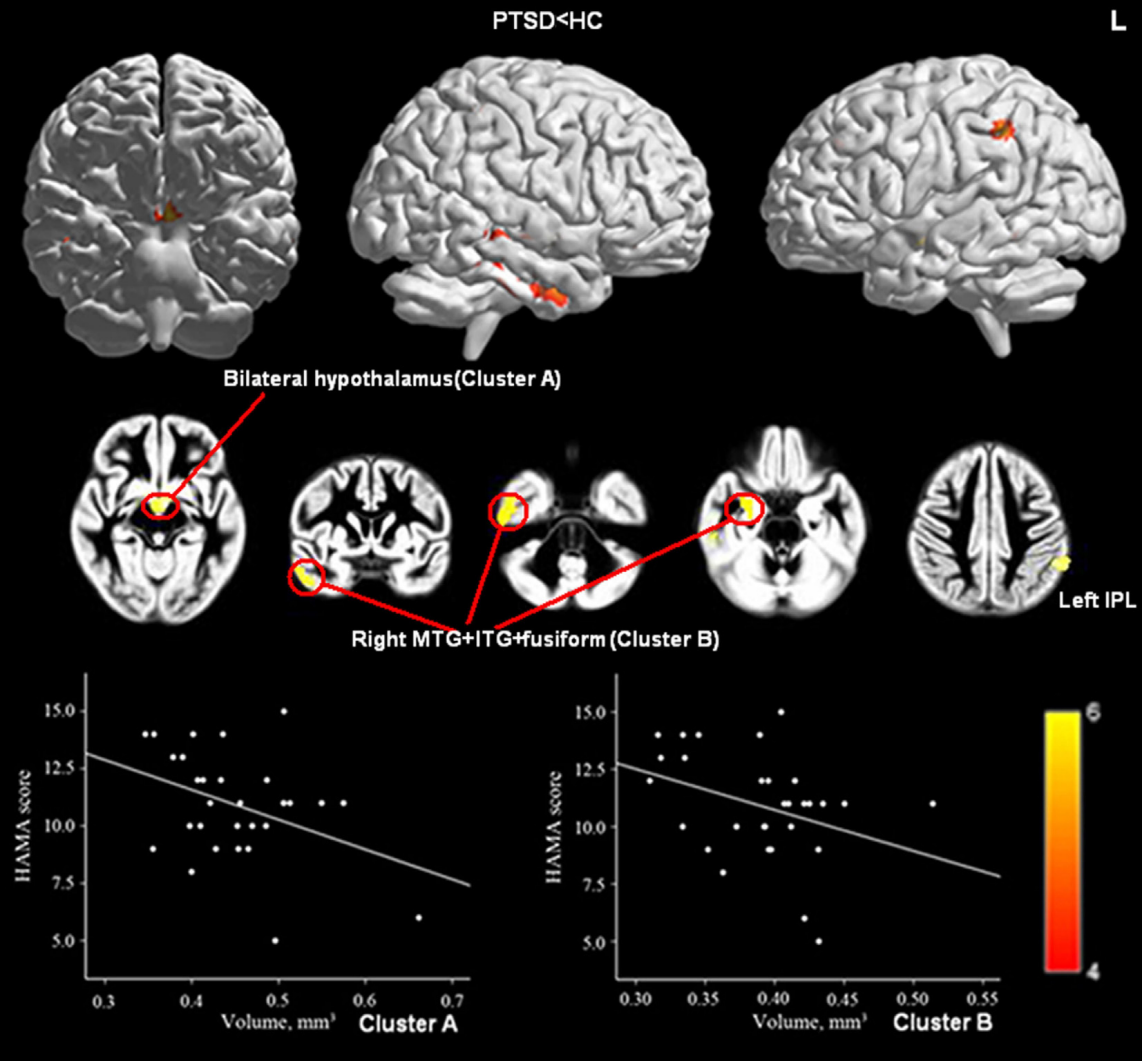
**Significant GMV decrease in patients with PTSD compared with controls (upper panel) and correlations between HAM-A scores and GMV of Cluster A (bilateral hypothalamus; Peak, x = −5, y = −4, z = −9, MNI) and Cluster B (right MTG, right ITG, and right fusiform gyrus; Peak, x = 53, y = −25, z = −23, MNI; lower panel)**. (*P*_cluster-level_ < 0.05 after FWE correction for whole-brain volume, age, and gender). Abbreviations: MTG, middle temporal gyrus.

Relative to HCs, the PTSD group showed reduced GMV in bilateral hypothalamus and left IPL as well as in a cluster coinciding with right middle temporal gyrus (MTG), right inferior temporal gyrus (ITG), and right fusiform gyrus (Figure 2; Table 3).

**Table 3:** ***Post hoc* whole-brain analysis of GMV differences between groups**.

Anatomical region	Location	Cluster size	Test value		MNI coordinate		
			*p*_(cluster-level)_	T			
**HC > PTSD**							
Hypothalamus	Bilateral	355	0.001	5.92	−5	−4	−9
IPL	Left	341	0.001	5.68	−54	−45	40
ITG+MTG+fusiform	Right	247	0.003	5.38	53	−25	−23
**OCD > PTSD**						
OFC	Left	632	<0.001	5.55	−20	20	17
CPL	Left	208	0.004	5.5	−53	−61	−29
PHG+OFC	Right	713	<0.001	5.41	29	11	−26
MTG+ITG	Left	496	<0.001	5.29	−54	−58	−5
IPL	Left	394	0.001	5.19	−62	−51	36
Fusiform+ITG	Right	302	0.002	4.94	54	−17	−30
MTG	Left	118	0.008	4.93	−53	−15	−18
**SAD > PTSD**							
OFC	Right	133	0.007	5.33	39	39	−20
MFG	Left	317	0.002	5.24	−45	26	42

*p_(FWE-corrected)_ < 0.05*.

*HC, healthy control; ITG, inferior temporal gyrus; MTG, middle temporal gyrus; PHG, parahippocampal gyrus; OFC, orbitofrontal cortex; MFG, middle frontal gyrus; CPL, cerebellum posterior lobe*.

### OCD versus PTSD

**Figure 3:**
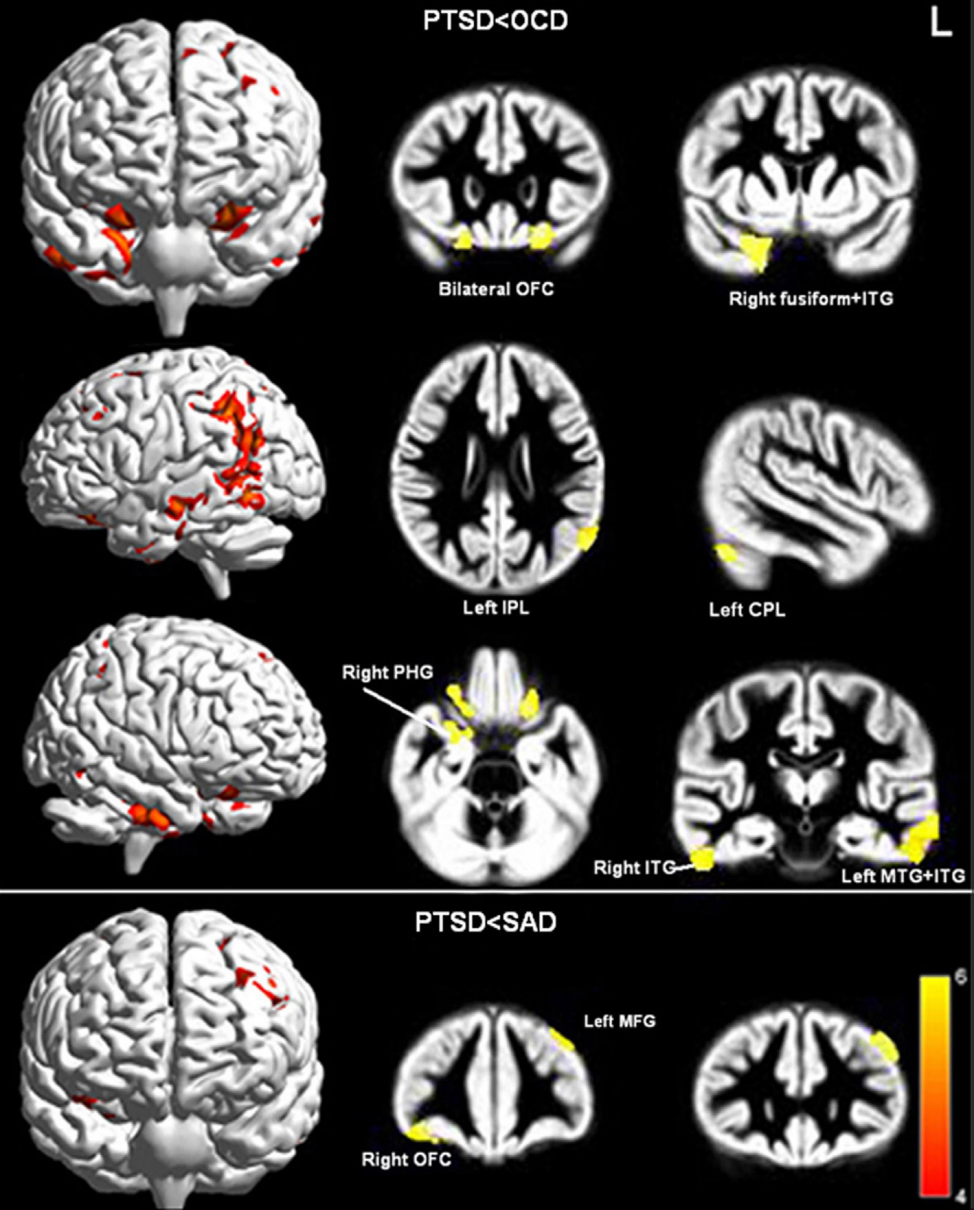
**Significant GMV decreases in PTSD compared to OCD (upper panel) and SAD (lower panel; *P*_cluster-level_ < 0.05 after FWE correction for whole-brain volume, age, gender, and illness duration)**. Abbreviations: OCD, obsessive–compulsive disorder; PTSD, post-traumatic stress disorder; SAD, social anxiety disorder; ITG, inferior temporal gyrus; MTG, middle temporal gyrus; PHG, parahippocampal gyrus; OFC, orbitofrontal cortex; MFG, middle frontal gyrus; CPL, cerebellum posterior lobe.

Relative to the OCD group, the PTSD group displayed reduced GMV in bilateral OFC, left cerebellum posterior lobe (CPL), bilateral middle temporal gyri (MTG), bilateral inferior temporal gyri (ITG), right parahippocampal gyri (PHG), left IPL, and right fusiform gyrus (Figure 3; Table 3).

### SAD versus PTSD

Relative to the SAD group, the PTSD group displayed reduced GMV in the right OFC and left middle frontal gyrus (MFG) (Figure 3; Table 3). No significant differences were evident with regard to GMV between the OCD/SAD groups and the HCs.

### Clinical correlation results

When patients from all three anxiety groups were combined, GMV in left hypothalamus was significantly negatively correlated with HAM-A score and showed a trend to correlate negatively with HAM-D scores (*p* = 0.052) (Peak, *x* = −5, *y* = −4, *z* = −9, MNI; Figures 1C,D). Furthermore, of the six ROIs extracted from the regions with reduced GMV in the PTSD group compared with the HCs, GMV of Cluster A (bilateral hypothalamus; Peak, *x* = −5, *y* = −4, *z* = −9, MNI) and Cluster B (right inferior/MTG and fusiform gyrus; Peak, *x* = 53, *y* = −25, *z* = −23, MNI) were negatively correlated with patient’s HAM-A scores (Figure 2). No significant correlations were evident for GMV in these regions between PCL, CAPS score, or disease duration.

## Discussion

To the best of our knowledge, the present study provides the first direct comparison of potential neuroanatomical differences in GMV between patients with PTSD, OCD, SAD, and HC using a whole-brain morphometric method. We find that the PTSD group of patients exhibit regional GMV differences not only compared to the HC group but also compared to the OCD and SAD groups of patients. In particular, GMV in left hypothalamus and IPL differed significantly among all participant groups (Figures 1A,B) and a *post hoc* analysis demonstrated that these GMV differences are primarily due to differences between the PTSD group and the other groups (Figure 1).

The hypothalamus is a key component of the hypothalamic–pituitary–adrenocortical (HPA) axis, which is a major part of the neuroendocrine system that controls reactions to stress (Teicher et al., 2002) and regulates many physiological processes, including digestion, mood, and emotions (Groenink et al., 2002). This HPA axis is a potential source of vulnerability with regard to stress-related psychopathology (Yehuda, 2002). For example, a number of preclinical studies suggest that early life stress induces long-lived hyper(re)activity of corticotropin-releasing factor (CRF) systems as well as alterations in other neurotransmitter systems, resulting in increased stress responsiveness (Heim and Nemeroff, 2001) and exaggerated glucocorticoid response to subsequent stressors (Bremner et al., 1997). Moreover, the hypothalamus is reported to play an important role in stress regulation, and to modulate the effect of the endocrine system on behavioral reactions (Kruk et al., 1998). Traumatic stressors tend to induce an increased release of cortisol, and this irregular hormonal response to stress might predispose a person toward developing PTSD (Miller et al., 2007).

A recent meta-analysis revealed that the HPA axis is abnormal in patients with PTSD (Klaassens et al., 2012) which is consistent with our finding of reduced GMV in the left hypothalamus and negatively correlated with anxiety symptoms (Figures 1A,C,D). These findings suggest that understanding the role of the hypothalamus in stress regulation could help to elucidate the neurobiological underpinnings of traumatic stress-related mental disorders. Only in patients with PTSD, was GMV of bilateral hypothalamus negatively correlated with HAM-A scores (Figure 2), which suggests that either the function of the hypothalamus might be more closely associated with anxiety symptoms in PTSD than in other disorders or the HPA is more disrupted in PTSD.

Inferior parietal lobule is an important region of parietal cortex which has been shown to be important for modulation of arousal (Heilman, 1997) and negative emotional processing (Etkin and Wager, 2007), and has been reported to by other researchers to be affected in PTSD (Heilman, 1997). Structural abnormalities of the IPL would most likely impair normal emotional control in patients with PTSD and this suggestion is consistent with previous studies in which it is reported that the IPL may be activated by observation of traumatic cues by HCs (Bremner et al., 2003) and during fear versus neutral conditions among patients with PTSD (Park et al., 2002). Thus, the reduction of the GMV in IPL that we observe in the PTSD group is both consistent with a potential role for this brain region in subserving the fear response and provides novel neurobiological evidence for the dysfunction of the IPL in patients with PTSD.

Interestingly, patients with PTSD exhibited greater structural differences than those with OCD versus those with SAD (Figure 3). These reduced GMV regions were primarily located in the prefrontal-temporal-limbic circuit (Figure 3). The large etiologic and symptomatic discrepancies between patients with PTSD and those with OCD might partially explain this result, whereas PTSD and SAD might be associated via similar exaggerated fear responses (Etkin and Wager, 2007).

Although prefrontal cortex has consistently been implicated in PTSD (Bremner, 1999), the current study is the first to report greater reduction of GMV in prefrontal cortex in PTSD compared with other anxiety disorders. OFC is a brain region that is crucially important for understanding human behavior. OFC is anatomically connected to the limbic structures and prefrontal cortex, which mediates executive function. In addition to its role in emotion and reward, the OFC is also suggested to be involved in sensory integration, which includes integrating the affective values of reinforcement, decision making, and expectation (Kringelbach, 2005). Moreover, OFC dysfunction is associated with inappropriate emotional responses to trauma and behavioral responses to stimuli and has been suggested to play an important role in symptom formation in patients with PTSD (Liberzon and Martis, 2006).

We also observed that patients with PTSD showed a greater reduction in GMV in right PHG compared to patients with OCD. PHG, which is a part of limbic system, plays an important role in memory encoding and retrieval. A previous fMRI study confirmed that patients with PTSD show functional alterations in parahippocampal regions and the prefrontal cortex during associative learning and memory (Werner et al., 2009). Thus, alterations in the structure of prefrontal-limbic networks might affect the memory functions of patients with PTSD.

As well as processing primary auditory, speech, and vision, temporal cortex is also involved in emotion and memory regulation. Specifically, fMRI findings have provided clear evidence that the ITG/fusiform gyrus integrates perception and memory (Bremner et al., 2003), is involved in accessing memory traces of past experiences of fear (Rolls, 2003) and other higher cognitive functions (Andreasen et al., 1995). On the other hand, it has been shown that hypoactivity of MTG is associated with impaired attention and memory among patients with PTSD (Bremner et al., 2003). Furthermore, an increasing body of evidence suggests that the functions of the ITG and the fusiform gyrus are associated with other cortical regions such as OFC, amygdala, hippocampal system, and prefrontal cortex (Rolls, 2003). Furthermore, the aforementioned regional defects in the temporal cortex of PTSD patients were correlated with anxiety symptoms in the current study (Figure 2). Combining the results of MRI and ethological studies (Rolls, 2003), might illuminate how impaired prefrontal-temporal-limbic networks influence higher brain functions and impair perception and emotion as well as long- and short-term memory among patients with PTSD.

Patients with PTSD also exhibited reduced GMV in left CPL compared to patients with OCD. In addition to motor functions, the cerebellum has been recently implicated in attention control and other cognitive functions (Villanueva, 2012). Furthermore, the cerebellum might be associated with the pathophysiology of PTSD (De Bellis and Kuchibhatla, 2006). The CPL of patients with mental disorders has previously displayed decreased regional activation (Baillieux et al., 2010), signal processing, and storage, all of which are relevant to auditory–verbal memory functions. Thus, the abnormal structure of the CPL found in the current study supports its neural involvement in the emotional regulation and traumatic memories associated with PTSD.

Compared with HCs, patients with PTSD displayed reduced GMV in several cerebral regions including bilateral hypothalamus, left IPL, and right temporal cortex (Figure 2). Furthermore, GMV in bilateral hypothalamus and right temporal lobe was negatively correlated with HAM-A scores (Figure 2). The fact that perceptual abilities are located in temporal cortex and the correlation between this brain region and PTSD symptoms might explain why traumatic experiences are required for this condition to develop (Friedman et al., 2011).

Taken together, impairments to the aforementioned brain regions might provide structural evidences for the mental abnormalities of PTSD patients, which might be caused by a combination of traumatic stress and psychopathological symptoms. Furthermore, different degrees of abnormality in the prefrontal-temporal-limbic circuit might explain the symptom differences between patients with PTSD, OCD, and SAD with regard to episodic memory, emotional processing, and executive control.

Methodological differences might explain why significant GMV differences were not evident in the OCD or SAD groups compared to the HCs in the current study. Our results were presented with a stricter FWE correction than has been applied in previous studies. When we set the statistical threshold to *p* < 0.001 without correction, our results indicated GMV differences between patients with OCD, SAD, and HCs. In addition, this strict threshold selection may also be the reason for non-significant GMV difference in some brain regions such as amygdala and hippocampus which have been widely reported to be affected in PTSD patients (Woon and Hedges, 2008). For example, when we repeated the analysis using an FDR correction, we find that the PTSD group does indeed exhibit a smaller volume in both right amygdala and right hippocampus compared to HC.

The direct comparison of cerebral GMVs between patients with different anxiety disorders using a whole-brain voxel-based analysis is challenging and the current study also bears several limitations. First, patients were recruited to the present study without considering group differences in illness duration, medication, and the severity of symptoms (HAM-A and HAM-D scores). Nevertheless, illness duration and the severity of symptoms were included as covariates through out the statistical process. Furthermore, the OCD patients who had received previously received drug treatment experienced a 2-week washout period before undergoing MR scanning so that confounding factors were actually relatively well controlled for in current study. Second, the sample sizes for each group although appreciable are still relatively small and this adds to the difficulty in obtaining statistical significant results. Future studies including recording of symptom severity and illness duration in well-matched drug-naïve, patients with larger sample sizes are warranted to more fully elucidate the neural bases of anxiety disorders and to firmly establish biomarkers to support differential diagnoses.

This study is the first to directly compare GMV across the entire brain among three different anxiety-related disorders, and this approach is highly consistent with recent trends in modern psychiatry in which brain imaging is being used to reveal the neural substrate of various psychiatric disorders (Insel and Cuthbert, 2015). By using a state of the art VBM-DARTEL whole-brain analysis, we found that patients with PTSD showed significant consistent reductions in GMV compared with patients with OCD and SAD, while the latter two groups of patients showed no significant differences from each other. These findings provide an important foundation for developing potential biomarkers to help distinguish between different psychiatric disorders. Although all patients were enrolled in this study under the DSM-IV system, this investigation nevertheless provides important information to develop brain imaging hypotheses to investigate the clinical significance of the advances in neuroscience and genetics of psychiatric illness incorporated in DSM-5 (Kupfer et al., 2013).

## Author Contributions

All authors have contributed to and have approved the final manuscript. QG and XH took the main responsibility for study design, initiating, and finally approving the version to be published. BC contributed to study design, data acquisition, data analyses, and writing this manuscript. SL, XH, YL, XW, and XY were responsible for data acquisition and recruitment of participants. CQ, YY, WZ, FB, and NR contributed to study design and editing the manuscript.

## Conflict of Interest Statement

The authors declare that the research was conducted in the absence of any commercial or financial relationships that could be construed as a potential conflict of interest.

## Footnotes

^1^http://www.fil.ion.ucl.ac.uk/spm/

^2^http://www.mccauslandcenter.sc.edu/mricro/index.html

^3^http://marsbar.sourceforge.net/
